# Inhibiting EGFR Dimerization Using Triazolyl-Bridged Dimerization Arm Mimics

**DOI:** 10.1371/journal.pone.0118796

**Published:** 2015-03-19

**Authors:** Laura E. Hanold, Krishnadev Oruganty, Norman T. Ton, Aaron M. Beedle, Natarajan Kannan, Eileen J. Kennedy

**Affiliations:** 1 Department of Pharmaceutical and Biomedical Sciences, College of Pharmacy, University of Georgia, Athens, Georgia, United States of America; 2 Department of Biochemistry and Molecular Biology, University of Georgia, Athens, Georgia, United States of America; Cornell University, UNITED STATES

## Abstract

The epidermal growth factor receptor (EGFR) is overexpressed in multiple carcinomas and is the focus of a variety of targeted therapies. Here we report the design of peptide-based compounds that mimic the EGFR dimerization arm and inhibit allosteric activation of EGFR. These peptides are modified to contain a triazolyl bridge between the peptide strands to constrain the EGFR dimerization arm β-loop. In this study, we demonstrate that these peptides have significantly improved proteolytic stability over the non-modified peptide sequence, and their inhibitory effects are dependent on the number of the methylene units and orientation of the introduced triazolyl bridge. We identified a peptide, EDA2, which downregulates receptor phosphorylation and dimerization and reduces cell viability. This is the first example of a biologically active triazolyl-bridged peptide targeting the EGFR dimerization interface that effectively downregulates EGFR activation.

## Introduction

**Fig 1:**
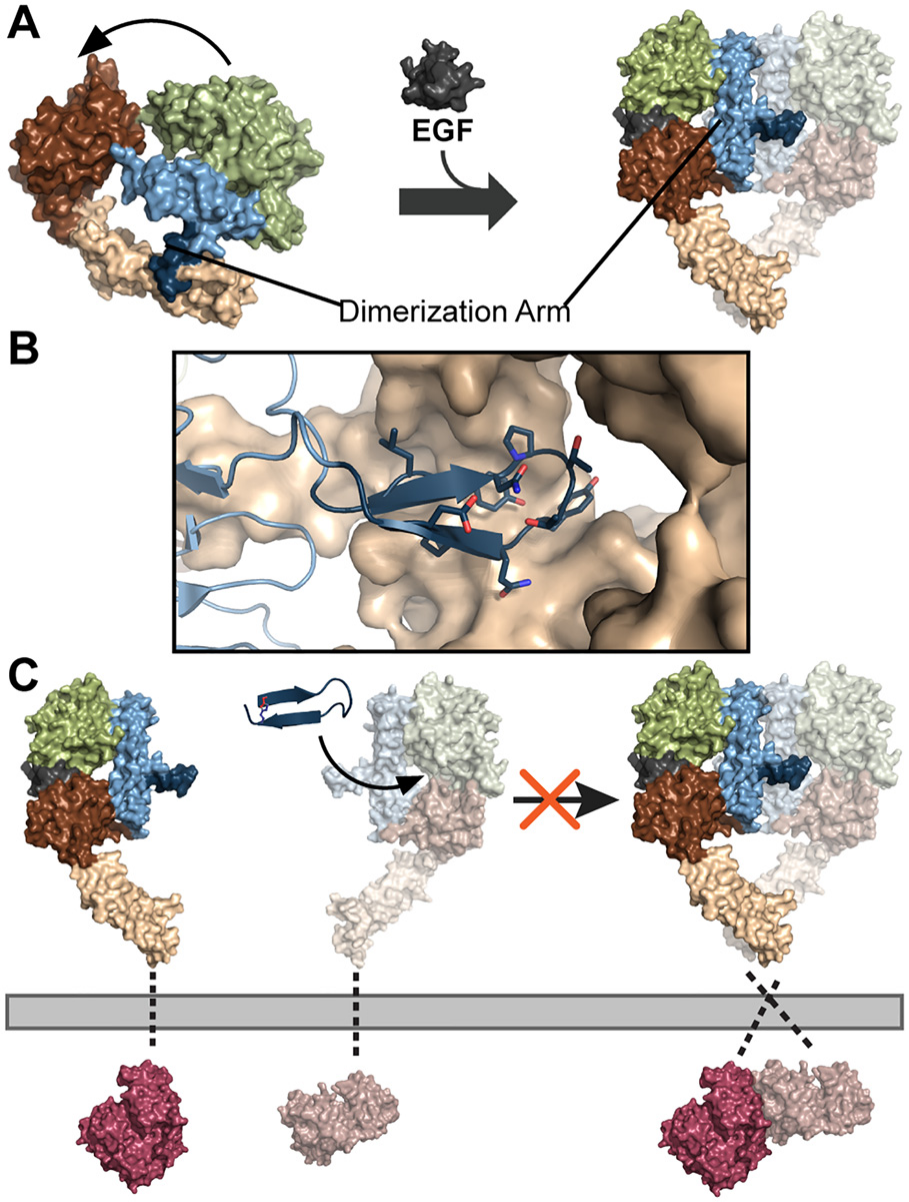
Dimerization arm targeting strategy for inhibition of EGFR. (a) EGF-induced activation of the extracellular receptor of EGFR. In the inactive state, the dimerization arm (dark blue) of the extracellular receptor is buried within domain IV (beige). In the active state, the receptor undergoes a conformational change to promote intermolecular interactions of the dimerization arm for receptor dimerization. Other features of the receptor include domain I (green), domain II (blue), domain III (brown) and EGF (dark grey). (b) The dimerization arm (dark blue) makes extensive contacts with domain II of the receptor binding partner (beige). (c) Triazolyl-bridged peptides were designed to mimic the dimerization arm, thereby blocking receptor dimerization and activation of the kinase (violet) through occlusion of the dimerization arm binding pocket. Additional features include the transmembrane domain (grey dashed lines). Structures were rendered using PyMol (PDB files: 1NQL, 3NJP, and 2GS6).

The Epidermal Growth Factor Receptor (EGFR) is a transmembrane receptor tyrosine kinase and member of the ErbB receptor family that performs key roles in cell regulation, including proliferation and differentiation [1]. As such, tight regulation of EGFR activity is essential to normal cell growth and function. There are several factors involved in EGFR regulation including ligand binding, conformational changes, dimerization, kinase activation, and internalization for downregulation, degradation or recycling [2–7]. Upon ligand binding, the extracellular receptor portion of EGFR undergoes considerable conformational changes between the inactive and active states [2,3]. In its inactive form, the receptor is folded so as to bury the dimerization arm. Once activated, EGFR undergoes a significant rearrangement that projects the dimerization arm outward to engage in receptor dimerization (Fig. 1). Dimerization of the extracellular receptor is largely dependent on dimerization arm interactions, and this allosteric change is followed by intracellular kinase domain dimerization and phosphorylation [2–6]. The phosphorylated tyrosine residues of the active kinase domain serve as docking sites for downstream proteins and promote signaling cascades involved in cell growth, proliferation, and migration. As an additional layer of regulation, the receptor can be internalized and degraded or recycled back to the membrane for continued signaling.

In addition to EGFR homodimerization, there are alternative modes of receptor oligomerization contributing to regulation of the EGFR signaling pathway, including heterodimerization, formation of ligand-free inactive dimers, and formation of higher order oligomeric clusters [8–12]. The various modes of oligomerization contribute to EGFR regulation and signaling complexity and may prime EGFR for ligand binding, provide spatial regulation for EGFR signaling, diversify signaling, and promote internalization of EGFR [10–13]. While it is known that these oligomeric structures can form, little is known about allosteric regulation governing some of these complexes. Thus, disruption of these various protein-protein interaction interfaces is necessary in order to evaluate their role in EGFR signaling. Since the vast majority of EGFR inhibitors target either the EGF binding site or the active site of EGFR [14,15], we sought to develop ligands that directly disrupt the dimerization interface. Previous studies showed that the dimerization arm of EGFR forms a large part of the dimer interface and contributes a substantial share of the driving energy for dimerization of the extracellular receptor (Fig. 1) [2,16]. The dimerization arm is a promising target for the design of ErbB disruptors and has been validated by the development of various compounds including pertuzumab, a monoclonal antibody that targets the dimerization arm of ErbB2, as well as a peptide dendrimer that targets this site on EGFR [17,18]. Additionally, an unconstrained peptide mimicking the ErbB3 dimerization arm and a disulfide-bridged peptide mimicking the EGFR dimerization arm were both shown to inhibit EGFR dimerization and phosphorylation [19–21]. However, non-modified peptides are inherently unstable to proteases, and disulfide bonds are sensitive to redox conditions and may become reduced in the acidic tumor microenvironment or endosomal compartments where EGFR signaling may occur [7,22,23]. As an alternative approach, we sought to introduce a covalent crosslinker into a dimerization arm mimic as a strategy to inhibit dimerization and downregulate EGFR activation.

Triazole crosslinks have been introduced into peptide-based scaffolds for diverse purposes. Previous work includes incorporating triazoles into peptide backbones or side chains [24] so as to either cyclize peptides [25–27], serve as the turn residues in β-turn mimics [28,29], replace disulfide bonds within β-hairpin structures [30], or to mimic β-strand configurations [31,32]. However, this chemistry had not previously been applied to the cyclization and stabilization of EGFR dimerization arm mimics. Thus, we sought to incorporate a triazolyl-bridge to covalently link the β-strands of the dimerization arm in an effort to improve the stability and inhibitory properties of the peptide mimic.

## Results and Discussion

### Peptide Design

**Fig 2:**
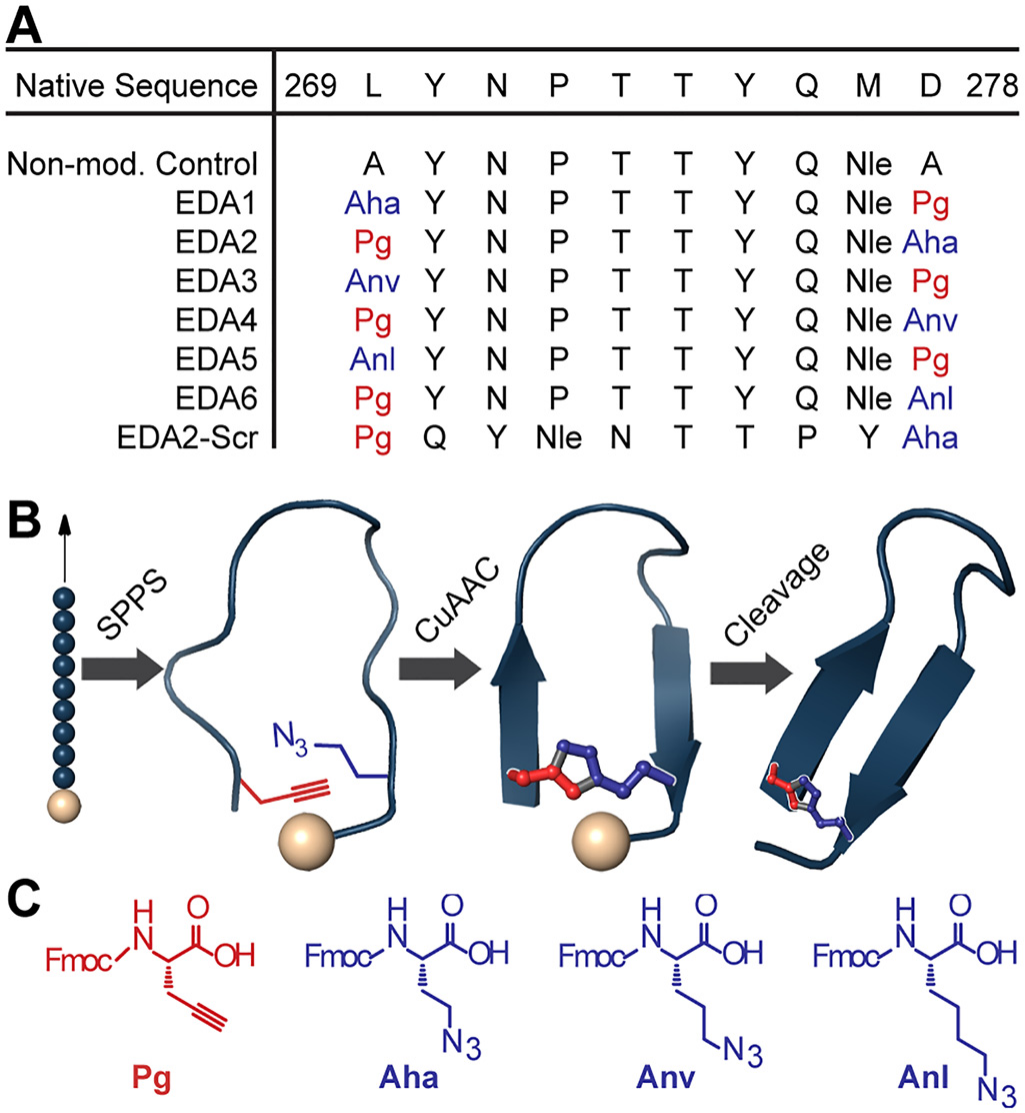
Design and synthesis of EDA peptides. (a) Peptide sequences were derived from the dimerization arm sequence of EGFR. The overall linker length and positioning of the azide and alkyne amino acids were varied. Non-natural amino acids are show in red and blue. (b) Dimerization arm mimics were synthesized by incorporating non-natural amino acids into the peptide sequence using solid phase peptide synthesis (SPPS). Peptides were cyclized on solid support via copper (I)-catalyzed azide-alkyne cycloaddition prior to resin cleavage. (c) Non-natural amino acids used for the triazole cross-link: N-Fmoc-L-propargylglycine (Pg), N-Fmoc-4-azido-L-homoalanine (Aha), N-Fmoc-5-azido-L-norvaline (Anv), N-Fmoc-6-azido-L-norleucine (Anl).

Since the dimerization arm plays a major role in the stabilization of the extracellular receptor dimer, multiple mimics were previously designed [17–21]. As an alternative strategy to covalently constrain the dimerization arm, we utilized cycloaddition chemistry to introduce a 1,4-disubstituted [1,2,3]-triazolyl-containing bridge between the terminal residues of the sequence. A panel of EGFR Dimerization Arm (EDA) peptides was designed using the native sequence of human EGFR (residues 269–278, Fig. 2a). The β-strand and turn residues were conserved from the original amino acid sequence since the majority of these residues make extensive contacts with the other receptor half-site.

Using this strategy, the dimerization arm was covalently cross-linked while on solid support to link the terminal residue side chains using copper (I)-catalyzed azide-alkyne [3+2] Huisgen cycloaddition chemistry (Fig. 2b) [24,33,34]. The azide- or alkyne-containing amino acids were incorporated into terminal positions of the sequence to minimize modifications within the dimerization arm itself. Since the optimal bridge length was not known, we modified this length by incorporating different azido-amino acid derivatives (Fig. 2c) that were synthesized as previously described [35]. The methylene units of the azido-amino acids were varied from 2 to 4 units (azido-L-homoalanine, azido-norvaline, or azido-norleucine) to alter the overall length of the triazole linker while the alkyne (propargylglycine) remained fixed. Since the linker asymmetrically connects the triazole, the peptides were synthesized in pairs by exchanging the positions of the azido- and alkynyl-amino acids. This was performed to evaluate the effects of the triazole position on inhibitory activity. In addition, two peptide controls were designed: one containing the non-modified sequence of the dimerization arm, and the other containing a scrambled sequence of the dimerization arm (Fig. 2a) [20].

### Molecular Dynamics Simulations of Triazolyl-Bridged Peptides

**Fig 3:**
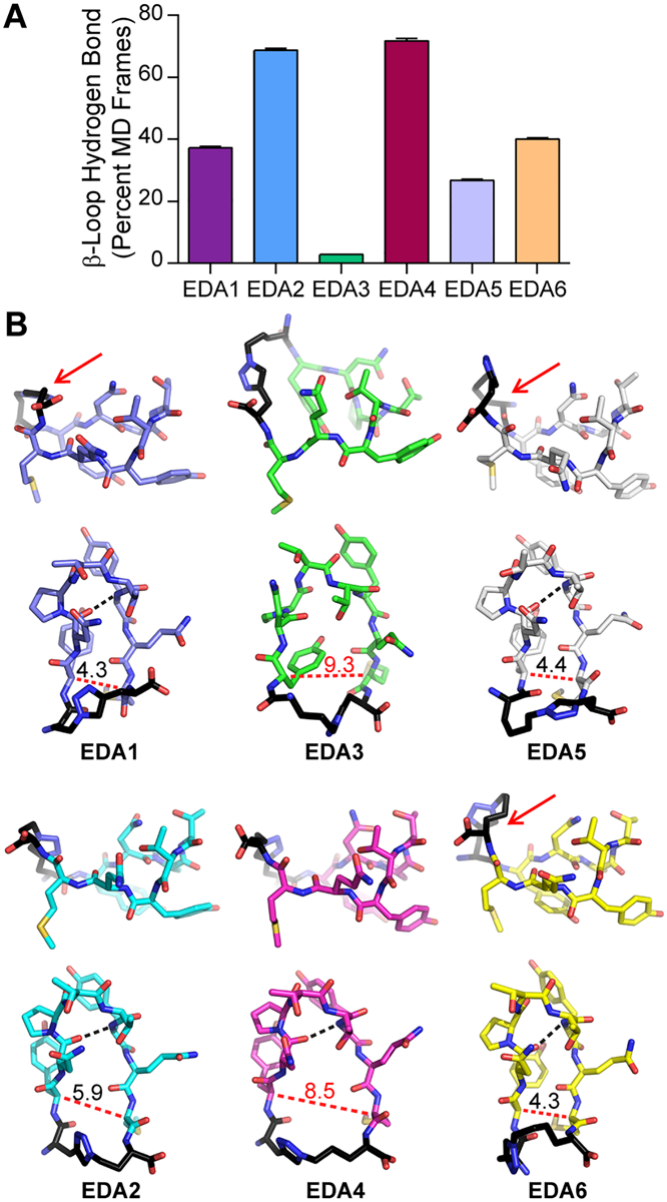
Molecular dynamics simulations of EDA peptides. (a) Molecular dynamics simulations were performed and the g_hbond program in Gromacs suite was used to determine the stability of the hydrogen bond characteristic of the β-loop conformation. A query of the number of frames in which the hydrogen bond between Asn271 and Tyr275 is present predicts that this hydrogen bond is largely maintained in EDA2 and EDA4. Data is plotted as the percent of molecular dynamics trajectory frames in which the hydrogen bond between Asn271 and Tyr275 is present. (b) Molecular dynamics simulations were performed to predict the overall structure of the EDA peptides and the cluster centers for EDA1–6 are shown. Red arrows indicate linkers that fold over the non-binding surface of the peptide. The black dashed line indicates the hydrogen bond between Asn271 and Tyr275. Widths between the β-sheets were measured between the Cα carbon of residues Tyr270 and Nle277 (red dashed line).

In order to predict the impact of the introduced triazolyl bridges on the overall structure of the dimerization arm, molecular dynamics simulations were performed. The effect of the linker length was studied in relation to the hydrogen-bonding network and the overall structure of the cyclized peptides (Fig. 3 and S1 Fig.). In the native structure, a hydrogen bond is present between Asn271 and Tyr275 and supports the β-loop structure. A query of the number of molecular dynamics (MD) frames containing the native H-bond for the triazolyl-bridged peptides predicts that EDA2 and EDA4 largely retain the hydrogen bond throughout the duration of the simulation, however, the H-bond is nearly absent in EDA3 and is only moderately retained in EDA1, EDA5 and EDA6 (Fig. 3a). This suggests that the structures of EDA2 and EDA4 may not significantly perturb the β-loop structure. However, cluster analysis predicts that EDA4 will adopt a more splayed conformation with a distance of 8.5 Å between the Cα carbon of Tyr270 and Nle277 of the terminal ends of the peptide strands (Fig. 3b), which is nearly twice the measured distance of 4.5 Å in the structure of the native sequence. On the other hand, EDA2 appears to have less perturbation to the β-loop conformation with a moderate width of 5.9 Å that more closely resembles the native structure. EDA1, EDA5 and EDA6 were found to resemble the crystal structure most closely by maintaining a β-loop conformation with widths of 4.3 Å, 4.4 Å and 4.3 Å, respectively, while also maintaining the β-loop hydrogen bond in approximately 30–50% of the MD frames. Further, the triazolyl bridge of EDA1, 5 and 6 appears to project outward from the β-strands and may cause steric hindrance with binding contacts on domain II of the receptor. On the other hand, the triazolyl bridge of EDA2 and EDA4 adopts a more planar conformation relative to the β-strands and may allow for more extensive contacts with the receptor surface. Additionally, EDA2 appears to have less conformational flexibility as only a single cluster was identified, whereas 2–3 clusters were identified for EDA4 and EDA5. Overall, the MD simulations demonstrate that EDA2 maintains the native H-bond to support β-loop formation, is not significantly perturbed in terms of distance between the β-strands, contains a triazolyl crosslink that does not significantly project outward, and has reduced conformational flexibility as indicated by cluster analysis. Taken together, the simulations suggest that the conformation of EDA2 is relatively stable as compared to the other peptides and may more closely mimic the native binding conformation of the dimerization arm.

### The Triazolyl-Bridge Enhances Peptide Stability

**Fig 4:**
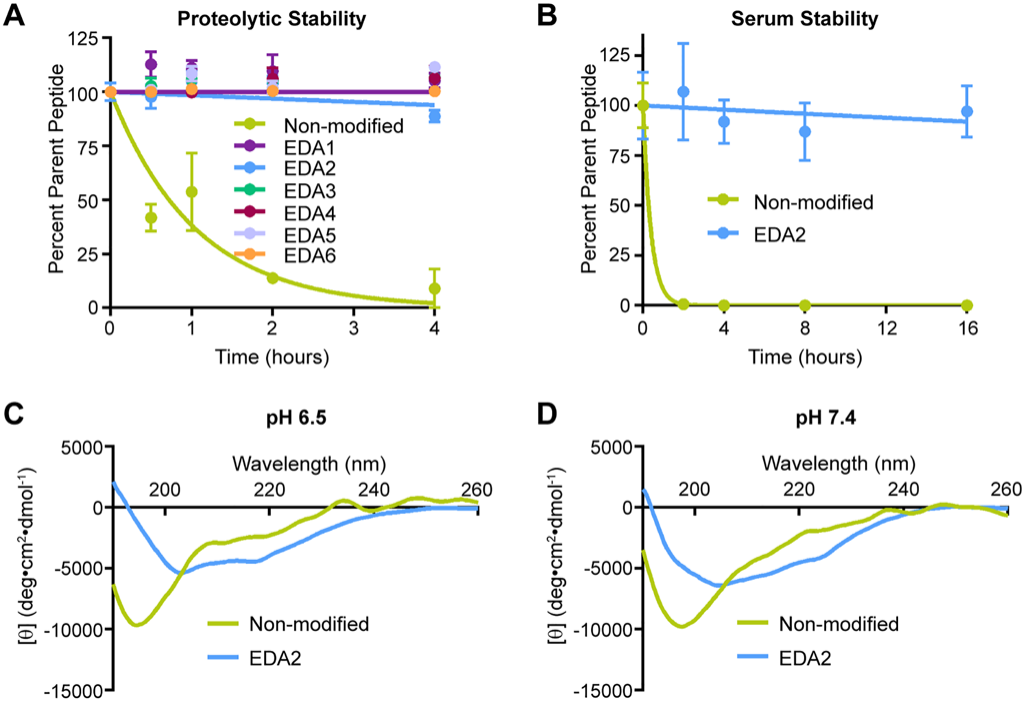
EDA peptides are resistant to proteolytic degradation. Proteolytic stability was measured in the presence of (a) a cocktail of immobilized chymotrypsin and trypsin over a time range of 0–4 hours and (b) 50% mouse serum over a time range of 0–16 hours. (c, d) CD spectra of the non-modified and EDA2 peptides were obtained on a Jasco J-710 CD Spectrometer at 25°C in 10 mM sodium phosphate buffer at pH 6.5 and 7.4. EDA2 maintains its structure under both conditions.

To determine whether the addition of a covalent constraint promoted proteolytic stability, degradation of the EDA peptides was measured in the presence of purified proteases, serum, and culture media (Fig. 4 and S2 Fig.). The rate of peptide degradation was first measured using purified proteases (Fig. 4a). EDA peptides were incubated with a cocktail of immobilized trypsin and chymotrypsin over a time course of four hours. The amount of remaining peptide was quantified by LC/MS relative to an internal standard. While the non-modified control peptide was rapidly degraded with 50% lost within one hour, all of the triazolyl-linked peptides showed significantly enhanced proteolytic resistance with little to no degradation over the 4-hour time course, demonstrating that introduction of the linker appears to provide substantial resistance to proteolytic degradation.

Peptide stability was also assessed using fresh mouse serum since multiple proteases are present in serum (Fig. 4b). Since there were not significant differences in stability between the panel of triazolyl-linked peptides and EDA2 was predicted to have minimal structural perturbations relative to the native dimerization arm, subsequent stability experiments were performed using EDA2 and a non-modified control. After incubation with plasma proteases over a sixteen-hour time course, the non-modified peptide was almost completely degraded within two hours, however, EDA2 showed nominal degradation during the entire time course tested. These results demonstrate that EDA2 is resistant to serum proteases over an extended time course. As another parameter of stability, peptide hydrolysis was also measured in tissue culture media (RPMI) over a four-hour time course (S2 Fig.). Both EDA2 and the non-modified control remained intact throughout the time course, illustrating that the peptide sequence is inherently stable in cell culture media beyond the duration of time courses used in subsequent cell-based assays.

Another physical parameter that was assessed was secondary structural characteristics at different pH values. This was addressed since the peptide may be exposed to acidic conditions such as the extracellular space of the tumor microenvironment and the endosomal compartments where internalized EGFR is sorted. Thus, structural stability of EDA2 and the non-modified control were measured at pH 7.4 and 6.5 using circular dichroism (Fig. 4c and d) [7,22,23]. Across both pH conditions tested, EDA2 largely retained its secondary structural characteristics as indicated by a broad minimum at approximately 205 nm. The non-modified control contains a more disordered structure with the representative minimum at approximately 195 nm. These results demonstrate that the secondary structure of EDA2 is not significantly altered between physiologic and slightly acidic pH conditions.

### Disruption of EGFR Phosphorylation and Cell Viability by EDA2

**Fig 5:**
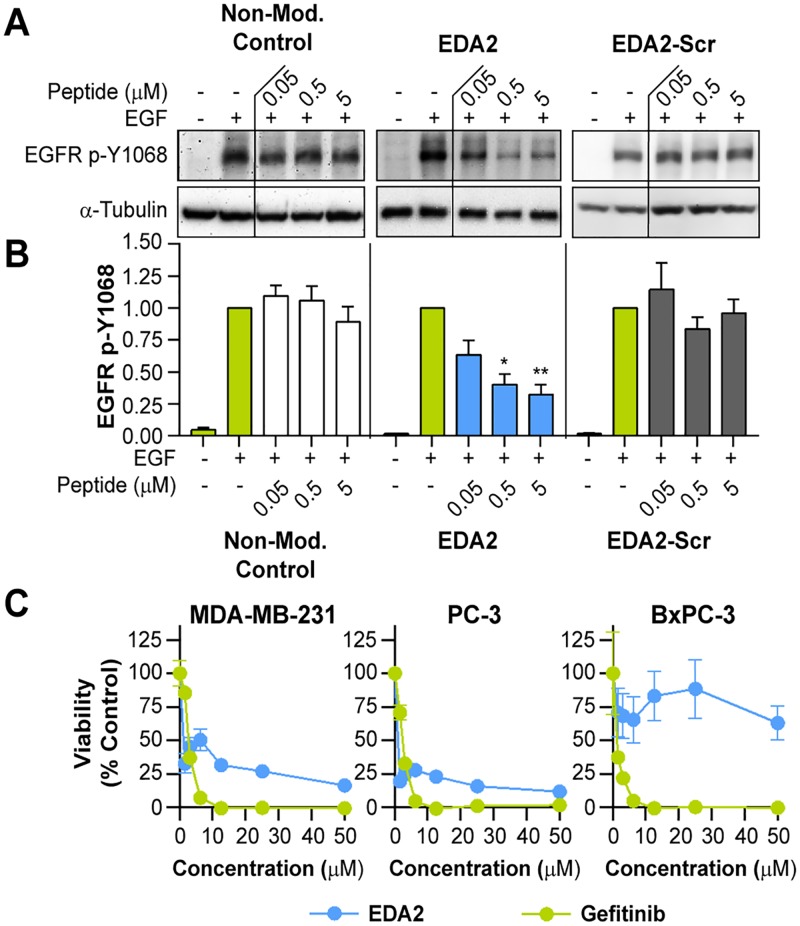
EDA2 down-regulates activated EGFR and reduces cell viability. (a) Cells were stimulated with 50 ng/mL EGF for 5 min in the presence or absence of EDA peptides, followed by western blotting. An apparent decrease in phosphorylated EGFR was observed when treated with EDA2. Vertical lines indicate non-adjacent samples from the same western blot. (b) Quantification of EGFR phosphorylated at Tyr1068, normalized to tubulin, shows that EDA2 reduces phosphorylated EGFR by greater than 60%, while the non-modified and scrambled controls do not. Data is plotted as the average of at least three experiments, where error bars represent SEM. * p < 0.05, ** p < 0.01 relative to the EGF-stimulated control. All remaining means are not significant (p > 0.05) relative to the EGF-stimulated control. (c) A panel of cell lines was dosed daily with EDA2 or gefitinib for 5 days, and viability was quantified using the Cell Titer Blue assay. EDA2 reduced viability by 50%, 72%, and 34% in MDA-MB-231, PC-3, and Bx-PC-3 cells, respectively. Data is plotted as the average of quadruplicates, where error bars represent SEM.

The EDA peptides were first tested to see if they could downregulate EGFR activation. Receptor activation was monitored as a function of EGFR autophosphorylation on Tyr1068 in intact MDA-MB-231 cells (Fig. 5a and b and S3 Fig.). The receptor was stimulated with EGF for 5 min following a 30 min peptide pretreatment. As predicted, EDA2 effectively downregulated EGFR activation while all the EDA peptides (EDA1 and EDA3–6) showed little to no activity (S3 Fig.). EDA2 was found to reduce EGFR phosphorylation by greater than 60% as compared to untreated cells at a dose of 5 μM (Fig. 5b). In contrast, the non-modified and EDA2 scramble (EDA2-Scr) controls had no inhibitory effect on EGFR phosphorylation, indicating that this effect is dependent on both sequence and cyclization. To further demonstrate that this effect was not due to changes in EGFR expression, an independent assay showed that total EGFR levels were not affected (S4 Fig.).

Since EDA2 appeared to downregulate EGFR phosphorylation, we sought to determine whether this activity could also lead to a decrease in cell viability (Fig. 5c). Three diverse, EGFR-expressing cell lines (MDA-MB-231, PC-3, and BxPC-3) were dosed daily with EDA2 over a 0–50 μM concentration range over a five-day time course, after which cell viability was quantified using the Cell Titer Blue assay. The small molecule inhibitor gefitinib was used as a viability control. At higher concentrations (10 μM or higher), viability was reduced to less than 25% in both MDA-MB-231 and PC-3 cells. At the lower dose of 6.25 μM, EDA2 reduced viability by 50% and 72% in MDA-MB-231 and PC-3 cell lines, respectively. On the other hand, BxPC-3 was less sensitive, where viability was only reduced by 34% at the same dose. Thus, while some cells display considerable sensitivity to EDA2 over the five-day period, others have less sensitivity and this may be due to additional mutational factors such as a mutant *TP53* gene in the case of BxPC-3 [36].

### EDA2 Disrupts EGFR Dimerization

**Fig 6:**
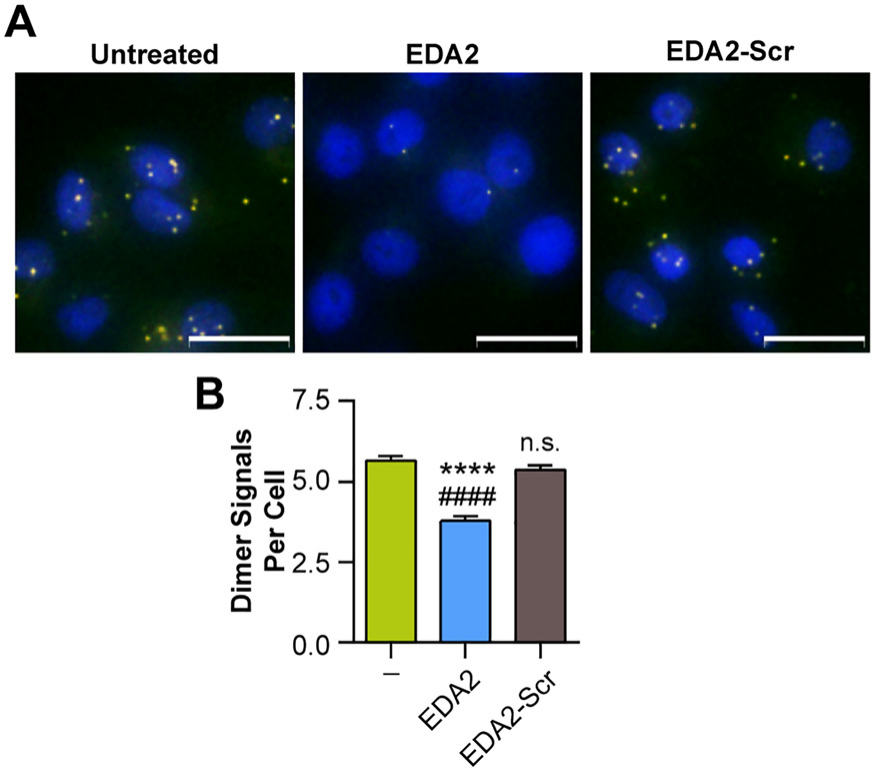
EDA2 down-regulates EGFR dimers. (a) MDA-MB-231 cells were stimulated with 10 ng/mL EGF for 5 min in the presence or absence of 5 μM EDA2 and the scrambled control peptide (EDA2-Scr). The dimer species of EGFR was detected using a fluorescent Duolink assay and is shown as an overlay of the PLA signal (orange) and DAPI (blue). When dimerized, one EGFR molecule may bind the plus probe while the other may bind the minus probe. The close proximity of the plus and minus PLA probes then allows for ligation and amplification, which can be detected as a punctate fluorescent signal. Images were obtained with a 40x objective and scale bars represent 25 μm scale. (d) The dimer signals of individual cells were measured for each condition tested (n = 500 cells per condition). Data is plotted as the average signal count per cell, where error bars represent SEM. **** p < 0.0001 relative to the stimulated control, #### p < 0.0001 relative to the scrambled control. Remaining comparisons do not differ significantly (p > 0.05).

While EDA2 downregulates receptor phosphorylation, we also wanted to investigate whether EGFR dimerization itself was inhibited. As a strategy to measure the number of detectable EGFR dimers in intact cells, a quantitative Proximity Ligation Assay (PLA) was performed (Fig. 6). Cells were stimulated with EGF in the presence or absence of EDA2 or its scramble control (EDA2-Scr). The cells were then fixed and probed with equivalent amounts of the plus and minus PLA probes conjugated to an anti-EGFR monoclonal antibody to illuminate EGFR dimers. Fluorescence microscopy was used to visualize the DAPI and PLA signals. EDA2-treated cells displayed a notable decrease in EGFR dimers, expressed as orange punctate signals, as compared to the untreated and EDA2-Scr control. Quantification of the fluorescent signals in individual cells (n = 500 cells) indicates that EDA2 caused a significant reduction in EGFR dimers by 33% (Fig. 6b). To verify that EDA2 did not simply reduce overall EGFR expression levels, total EGFR was measured under the conditions tested. EDA2 and the control peptides were found to have no effect on EGFR expression (S4 Fig.). Overall, these results show that EDA2 reduces EGFR dimerization and demonstrates the potential of EDA2 for downregulating EGFR activation and signaling.

## Conclusions

In summary, we developed a triazolyl-bridged peptide mimicking the EGFR dimerization arm. This peptide was found to have increased proteolytic resistance and maintained secondary structural characteristics in an acidic environment. EDA2 was further shown to downregulate EGFR dimerization, phosphorylation and cell viability. Although the native sequence of the dimerization arm was used in this study, this represents a starting point for sequence optimization so as to improve target inhibition and optimize binding interactions. These compounds can be used to probe homo- and heterodimerization of ErbB members. By targeting the dimer interface, analogous peptides can also be engineered to specifically target other members of the EGFR family so as to probe dimerization. Further, the peptides can be applied to study the effects of oligomerization on EGFR regulation. While peptide-based therapeutic candidates are generally characterized by high clearance and low bioavailability as compared to small molecules, chemical modifications may help to increase their half-life and uptake as demonstrated by stapled peptides currently in clinical trials [37–40]. The EDA peptides demonstrate resistance to proteolytic degradation, yet further improvements upon their ADME characteristics such as circulatory half-life may be achieved by additional modification such as PEGylation. Thus, further optimization of the EDA2 scaffold may lay the foundation for novel therapeutic treatment strategies of EGFR-overexpressing tumors. Ultimately, this study demonstrates novel utility of intrastrand triazole crosslinking to generate proteolytically stable, biologically active peptide-based macrocycles for targeted EGFR inhibition.

## Materials and Methods

### General Information

All protected amino acids, Rink amide MBHA resin and PAL-NovaPEGresin were purchased from Novabiochem unless otherwise noted. HCTU was purchased from Peptides International. All synthesis reagents and solvents were purchased from Fisher, Sigma-Aldrich, or Acros and used without further purification. Cell culture media and phosphate buffer saline were obtained from Lonza, fetal bovine serum from Thermo, penicillin/streptomycin and bovine serum albumin from Amresco, trypsin EDTA from Cellgro, and EGF from Abcam. Antibody to EGFR p-Y1068 was purchased from Abcam or Pierce, total EGFR from Santa Cruz Biotech, , and α-tubulin from the University of Iowa. PVDF membrane was purchased from Millipore. Immobilized chymotrypsin was obtained from Proteochem, and chemiluminescent substrate and immobilized trypsin were obtained from Pierce. Peptide characterization and purification were performed on a Zorbax SB-C18, 5 μm HPLC column using an Agilent 1200 series HPLC system coupled to the Agilent 6120 quadrupole LC/MS. Peptides and viability assays were quantified using the Biotek Synergy 2 microplate reader. C57BL/6J mice obtained from Jackson Laboratories were maintained in a local breeder colony. All mouse housing and handling procedures were approved by the University of Georgia Institutional Animal Care and Use Committee.

### Cell Culture

MDA-MB-231 cells purchased from ATCC were cultured in Roswell Park Memorial Institute-1640 (RPMI) with L-glutamine (Lonza) and supplemented with 10% fetal bovine serum (Thermo Scientific) and penicillin/streptomycin (Amresco). Cells were grown at 37°C with 5% CO_2_.

### Synthesis of Azido Amino Acids

Azido derivatives of amino acids were synthesized as previously described.[35] Fresh triflic azide was prepared immediately before the azido transfer reaction. Briefly, 1M solution of triflic anhydride in dichloromethane (DCM) (6 ml, 6 mmol, 3 equiv.) was added to a solution of sodium azide (0.781 g, 12 mmol, 6 equiv.) in 5 ml water at 0°C. The solution was stirred for 2.5 hours at 0°C, after which saturated sodium bicarbonate was added until the evolution of CO_2_ stopped. The solution was transferred to a separatory funnel and the organic layer was collected. The aqueous layer was washed two times with DCM. The organic layers were returned to the separatory funnel to be added dropwise to the amino acid.

An aqueous solution containing zinc chloride (18.5 mg, 0.14 mmol) and 1 equivalent (2 mmol) of either Fmoc-Dab-OH (Bachem), Fmoc-Orn-OH (Bachem) or Fmoc-Lys-OH (CHEM-IMPEX) was added to a roundbottom flask. The solution was stirred and triethylamine (0.837 ml, 6 mmol, 3 equiv.) was slowly added. Methanol (40 ml) was added dropwise to create a final ratio of water/methanol/DCM at 3:10:3. After complete addition of methanol, the freshly prepared triflic azide solution was added dropwise from the separatory funnel. The solution was stirred at ambient temperature for 2–3 hours and the reaction was monitored by TLC. The organic solvents were removed by rotary evaporation, and the remaining aqueous solution was acidified with 1% (w/v) citric acid (20 ml). The aqueous layer was extracted with DCM and the combined organic layers were purified by silica gel chromatography using a 0.5–7% (v/v) methanol gradient in DCM. Final products were confirmed by NMR and mass spectrometry.

### Peptide Synthesis

Peptides were prepared using Fmoc-based solid-phase peptide synthesis. The non-modified peptide control was synthesized on Rink amide MBHA resin. Peptides that were engineered to contain a triazole cross-link were synthesized on PAL-NovaPEG resin. Resin (25 μmol) was loaded into a fritted peptide synthesis column and equilibrated in N-methylpyrrolidinone (NMP). Deprotections were performed using a solution of 25% (v/v) piperidine in NMP. Amino acid couplings were performed using a 0.5 M solution of amino acid (0.5 ml, 0.25 mmol, 10 equiv) along with 0.5 M solution of HCTU (0.495 ml, 0.248 mmol, 9.9 equiv) and DIEA (87 μL, 0.5 mmol, 20 equiv). All couplings containing the azido-amino acids or propargyl glycine (Peptech) were performed using 4 equiv of 0.5 M amino acid (0.1 mmol, 0.2 ml), 3.96 equiv of 0.5 M HCTU (0.099 mmol, 0.25 ml), and 8 equiv of DIEA (0.2 mmol, 43.5 ml).

### On-Resin Cyclization of EDA Peptides

The 1,4-disubstituted-[1,2,3]-triazolyl cross-link was formed on-resin using copper(I)-catalyzed azide-alkyne cycloaddition chemistry. The resin (25 μmol) was suspended in a 1:2 solution of t-butanol (Alfa Aesar) and water. Sodium ascorbate (160 mg, 750 μmol, 30 equiv.) and copper sulfate (55 mg, 375 μmol, 15 equiv.) were added, causing the mixture to turn bright orange. The mixture was left to stir overnight. The mixture was then returned to a synthesis column, washed with 1:2 solution of t-butanol/water, and dried for a test cleavage. Cyclization was confirmed by a shift in retention time using LC/MS.

### N-terminal Fluorescein Labeling

Unless otherwise stated, all peptides used in this study were labeled with 5(6)-carboxyfluorescein (Acros) after on-resin cyclization or after the addition of β-Ala. After equilibrating the resin in DMF, 5(6)-carboxyfluorescein (19 mg, 50 μmol, 2 equiv), HCTU (19 mg, 45 μmol, 1.8 equiv) and DIEA (20 μL, 115 μmol, 4.6 equiv) were dissolved in 1 mL DMF. The solution was added to the resin and bubbled overnight while protected from light.

### Peptide Cleavage

Resin cleavage was performed by incubating the resin for 4–5 hours in a cleavage solution containing 95:2.5:2.5 trifluoroacetic acid/water/triisopropylsilane. After cleavage, the peptide was filtered through glass wool into ice cold methyl t-butyl ether. The precipitate was collected by centrifugation at 4°C. The supernatant was decanted and the pellet was air-dried. The peptide product was dissolved in methanol and products were confirmed by LC/MS. Non-modified control molecular weight = 1568.6 (expected 1569.62); Disulfide Control = 1632.6 (expected 1631.74); EDA1 = 1648.0 (expected 1648.68); EDA2 = 1648.0 (expected 1648.68); EDA3 = 1662.2 (expected 1662.71); EDA4 = 1662.2 (expected 1662.71); EDA5 = 1676.2 (expected 1676.74); EDA6 = 1676.2 (expected 1676.74); EDA2-Scr = 1647.6 (expected 1648.68)

The peptides were purified by reverse phase HPLC on a Zorbax SB-C18 column using a flow rate of 4 ml/min and a gradient of 10%–100% (v/v) acetonitrile containing 0.1% (v/v) TFA over 24 minutes. Fluorescein labeled peptides were quantified using the extinction coefficient of 68,000 L mol^-1^ cm^-1^ for fluorescein in 10 mM Tris pH 8.

### Modeling of EDA Peptides and Molecular Dynamics Simulation

The peptide coordinates corresponding to EDA wild-type were taken from the EGFR dimer structure 3NJP (residues 270–277). Avogadro was used to model the triazolyl linker between the terminal ends of the peptide before Tyr270 and after Met277. The geometry of the cross-linked peptide was minimized using the GAFF force-field implemented within Avogadro. The topological parameters, partial charges and other force field parameters were estimated with programs in the AmberTools 13 suite [41]. The amber generated force field values were converted to gromacs format using acpype [42]. Molecular dynamics simulations were carried out using Gromacs v4.6.2.

The peptide structures were solvated in a periodic dodecahedron box with at least 1nm space on all sides of the peptide. Energy minimization using steepest descent was carried out until Fmax reached 10 kJ/mol/nm. An NVT simulation with position restraints on the peptide atoms were performed with a V-rescale thermostat used for temperature equilibration. Long range electrostatics were treated using PME, with a short range cutoff of 0.9 nm. Pressure equilibration was done under similar conditions (including position restraints) with an additional Berendsen pressure coupling algorithm. Production runs for 40ns were carried out under the NPT conditions after removing the position restraints. The frames of the trajectory were saved to disk every 2ps.

The g_hbond program in the Gromacs suite was used to query the number of frames in which the hydrogen bond between Asn271 and Tyr275 was observed. The hydrogen bond between Asn271 and Gln276 was seen in all peptides with equal occupancy and hence is not discussed in the text. Cluster analysis of the trajectories was carried out with the g_cluster program in the Gromacs suite with a RMSD cutoff of 1.0 angstrom. The cluster centers were written to a PDB file and analyzed.

### Proteolytic Degradation of Peptides

Immobilized trypsin and chymotrypsin were added to 5 μg peptide in digestion buffer (100 mM Tris, 200 mM NaCl, 100 mM CaCl_2_, pH 8.2) where E/S for each protease was ~375. Benzyl alcohol was used as an internal standard. Peptides were incubated at 37°C for 0, 0.5, 1, 2 and 4 hours with gentle agitation, after which methanol was added to a final concentration of 50% (v/v). The immobilized protease was pelleted by centrifugation at 15,000 rpm, and the supernatant was collected and analyzed by LC/MS. After incubation with protease, the disulfide and triazole peptide spectra displayed a peak with a mass equivalent to the methylated peptide. Data represents the combined absorbance of both methylated and unmethylated peptides. Peptide absorbance at 220 nm was normalized relative to the benzyl alcohol peak. Data was plotted in GraphPad Prism as percentage of parent peptide relative to T0. Normalized curves represent one phase decay where Y0 equals 100, the plateau equals 0, and K is greater than 0.

### Peptide Stability in Tissue Culture Media

A solution of 0.2 mM peptide and 0.1% benzyl alcohol in RPMI-1640 supplemented with 0.1% (w/v) BSA was incubated at 37°C with mixing at 300 rpm. Aliquots were collected in duplicate and quenched with an equal volume of acetonitrile with 0.1% (v/v) TFA. The samples were centrifuged at 14,000 rpm and the supernatant was analyzed by LC/MS. The peptide absorbances at 220 nm were normalized relative the benzyl alcohol standard and the percent of remaining peptide was calculated relative to T0. The resulting data was plotted in GraphPad Prism.

### Serum Stability of Peptides

Blood for serum was collected from mice under the following University of Georgia Animal Use Protocols: A2010 08–153 and A2013 07–016. Fresh blood was obtained from isoflurane-anesthetized C57BL/6J male or female mice by terminal cardiac puncture according to standard procedures. Serum was separated from clotted blood by 5 min centrifugation at 10,000 g. A solution of 70% (v/v) fresh mouse serum, 0.1% (v/v) benzyl alcohol, and 0.2 mM peptide in PBS was incubated at 37°C in a thermomixer with shaking at 300 rpm. Aliquots were collected in duplicate at 0, 2, 4, 8 and 16 hours and quenched by the addition of an equal volume of acetonitrile and 0.1% (v/v) TFA. The proteins were pelleted by centrifugation at 14,000 rpm. The supernatant was collected and analyzed by LC/MS. Peptide and benzyl alcohol absorbance at 220 nm was recorded, and the percent parent peptide remaining was calculated relative to the benzyl alcohol internal standard. Data was plotted in GraphPad Prism as percent parent peptide relative to T0. Normalized curves represent one phase decay where Y0 equals 100, the plateau equals 0, and K is greater than 0.

### Circular Dichroism

CD spectra were obtained on a Jasco J-710 CD Spectrometer at 25°C using a 0.1 mm cuvette. Solutions of the non-modified control, disulfide control, and EDA2 in 10 mM sodium phosphate buffer at pH 6.5 and pH 7.4 were prepared. Spectra were recorded over 190–260 nm at 0.5 nm intervals using a 2 nm bandwidth, 100 ms time constant, 50 nm/min scanning speed, and 3 responses. The units were converted to molar ellipticity in Jasco Spectra Manager after baseline subtraction. The Savitzky-Golay smoothing filter was applied with a convolution width of 21.

### EGFR Phosphorylation in MDA-MB-231 Cells

MDA-MB-231 cells cultured on non-tissue culture-treated plates were seeded in 24 well tissue culture-treated plates in RPMI-1640 supplemented with 10% FBS and penicillin/streptomycin. Cells were incubated at 37°C until approximately 80% confluent. The media was then replaced with RPMI 1640 supplemented with 0.1% (w/v) BSA, and the cells were incubated overnight. Serum starved cells were then pretreated with 0.05, 0.5, or 5 μM peptide for 30 min, followed by a 5 min stimulation with 50 ng/mL EGF. Cells were immediately lysed in 1x Laemmli buffer.

Proteins were separated by 8% SDS-PAGE then transferred onto PVDF membrane. Identical membranes were probed for EGFR p-Tyr1068 and α-Tubulin. Bands were visualized by chemiluminescence and quantified using Licor Image Studio Lite. Results were normalized to the corresponding α-Tubulin and reported as a ratio relative to EGF-stimulated cells in the absence of peptide. The average of at least three experiments was plotted in GraphPad Prism, where error bars represent SEM. Significance values were calculated using two-way ANOVA with Bonferonni multiple-comparison test. Evaluation of total EGFR levels upon treatment with the non-modified peptide, EDA2, and EDA2-Scr was performed in separate experiments as described above. Total EGFR is reported as the average of two experiments performed in at least duplicate, where error bars represent SEM. Significance values were calculated using one-way ANOVA with Bonferonni multiple-comparison test.

### Cell Viability Assays

MDA-MB-231, PC-3, or Bx-PC3 cells were seeded at 7,500 cells/well in a 96 well plate and allowed to adhere in RPMI-1640 containing 1% FBS. Media was then replaced with RPMI-1640 containing 0.1% FBS and peptide or gefitinib at concentrations ranging from 1.56 to 50 μM. Treatment solutions were refreshed daily for a total of five days of treatment. After 5 days of treatment, the media was replaced with 100 μL serum free RPMI and 10 μL of Cell Titer Blue reagent (Promega) was added. Cells were incubated at 37°C for 5 hrs. Fluorescence was quantified using 530 nm excitation and 590 nm emission filters. Data is plotted in GraphPad Prism as the average percent viability relative to the vehicle control, where error bars represent SEM.

### EGFR Dimerization Duolink Fluorescence Assay

MDA-MB-231 cells were seeded into 8 well chamber slides in RPMI-1640 supplemented with 10% FBS and 1x penicillin/streptomycin. The cells were grown to 80–90% confluence then serum starved for 24 hours in RPMI-1640 supplemented with 0.1% (w/v) BSA. The cells were then treated with 5 μM peptide for 30 minutes and stimulated for 5 minutes with 10 ng/mL EGF. The cells were then washed with PBS and fixed with 2% paraformaldehyde and permeabilized in Triton-X 100. The Duolink In Situ Assay was performed according to the manufacturer’s instructions. Wells were blocked using the Duolink Blocking Solution. Equal volumes of the Plus and Minus PLA probes conjugated to the EGFR monoclonal antibody (Millipore) were added to the wells and incubated at 4°C overnight. The probes were then ligated and amplified using the Duolink Orange Detection Kit. The slide was mounted using Permafluor mounting medium with Dapi. Images were obtained at 40x magnification using TRITC and DAPI filters on an IX71 inverted fluorescent microscope with Cell Sens software (Olympus). Fluorescent signals per cell were counted from five representative 40X magnification microscope fields per condition. The averages from a single experiment performed in duplicate were plotted using GraphPad Prism. Error bars represent SEM. Significance values were calculated in GraphPad Prism using the D’Agostino-Pearson normality test, which demonstrated a non-Gaussian distribution, followed by a non-parametric ANOVA with Kruskal-Wallis and Dunn’s multiple-comparison tests.

## Supporting Information

S1 Fig RMSD plots for 40 ns simulations. The RMSD was calculated for all atoms in each peptide.
(TIF)

S2 Fig EDA2 is stable in tissue culture medium. Peptide stability was measured in the presence of RPMI-1640 tissue culture medium over a time range of 0–4 hours at 37°C. The relative amount of loss as compared to that of the parent peptide at t = 0 was quantified by LC/MS using an internal standard. Data is plotted in GraphPad Prism as the average of duplicates, where error bars represent SEM.
(TIF)

S3 Fig The effect of EDA1 and EDA3–6 on EGFR phosphorylation in MDA-MB-231. Serum starved MDA-MB-231 cells were treated with peptide or vehicle for 30 minutes, after which cells were stimulated for 5 minutes with 50 ng/mL EGF. Cells were immediately lysed following stimulation and proteins were separated by 8% SDS-PAGE. Western blot analysis showed that EDA1 and EDA3–6 do not inhibit EGFR phosphorylation at Tyr1068. Vertical lines indicate non-adjacent samples from the same western blot. Data is plotted as the average of three experiments, where error bars represent SEM. All peptide means did not significantly differ (p > 0.05) from the EGF-stimulated control.
(TIF)

S4 Fig The effect of EDA2 and its controls on total EGFR protein levels in the cell. (a) Serum starved MDA-MB-231 cells were treated with peptide or vehicle for 30 minutes then stimulated with 50 ng/mL EGF for 5 minutes. Cells were immediately lysed and total EGFR levels were analyzed by western blotting. (b) Total EGFR was quantified and normalized to the tubulin loading control. Data is plotted as the average of two experiments performed in duplicate and triplicate, where error bars represent SEM. The means did not significantly differ (p > 0.05), indicating that the peptides do not affect total EGFR protein levels in the cell.
(TIF)
