# Supplementary figures and images for: Inhibiting EGFR Dimerization Using Triazolyl-Bridged Dimerization Arm Mimics
Source: PLoS One. 2015 Mar 19;10(3):e0118796. doi: 10.1371/journal.pone.0118796 (PMC4366150; doi:10.1371/journal.pone.0118796)

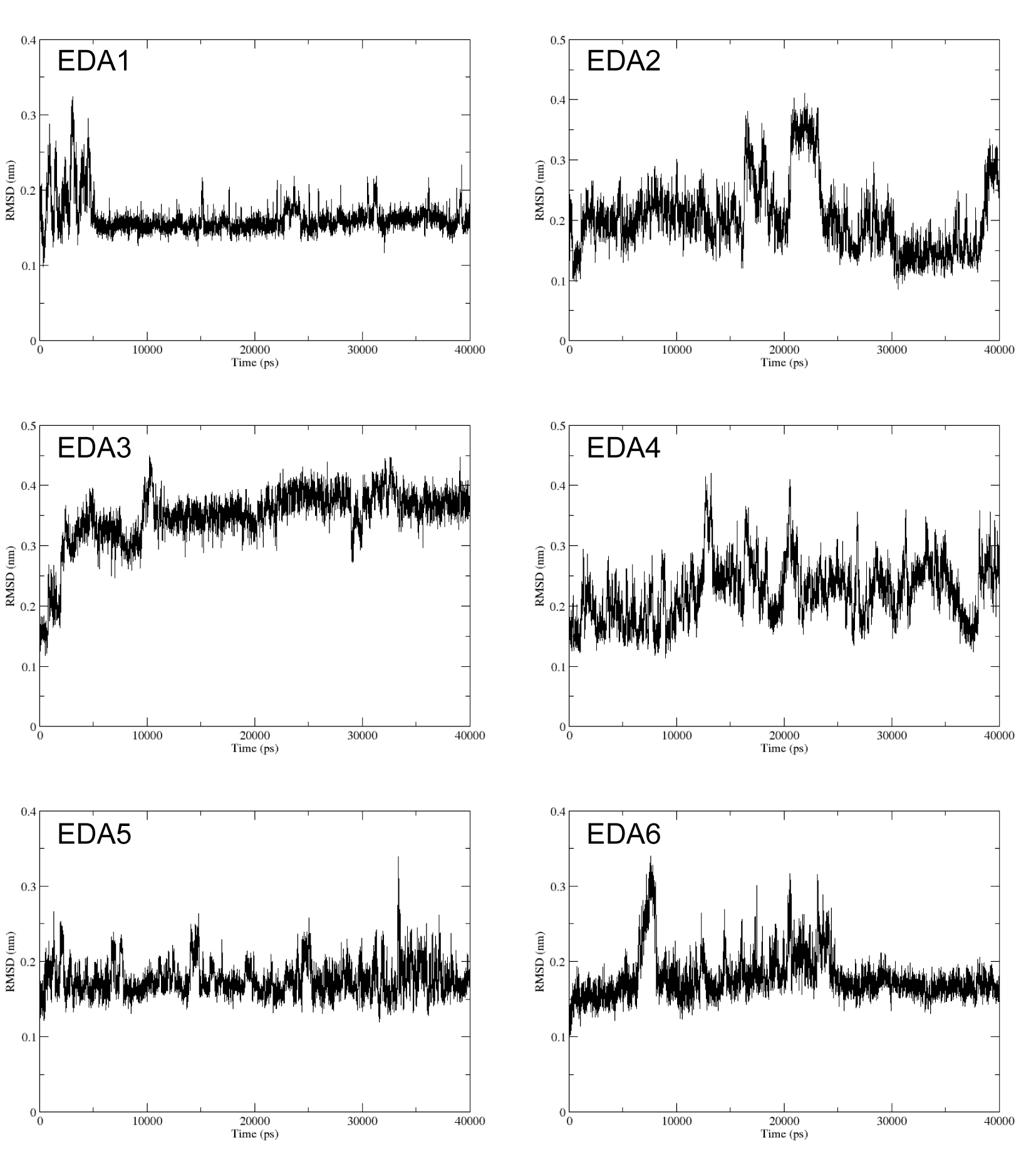

Supplement: S1 Fig — The RMSD was calculated for all atoms in each peptide. (TIF) [file pone.0118796.s001.tif]

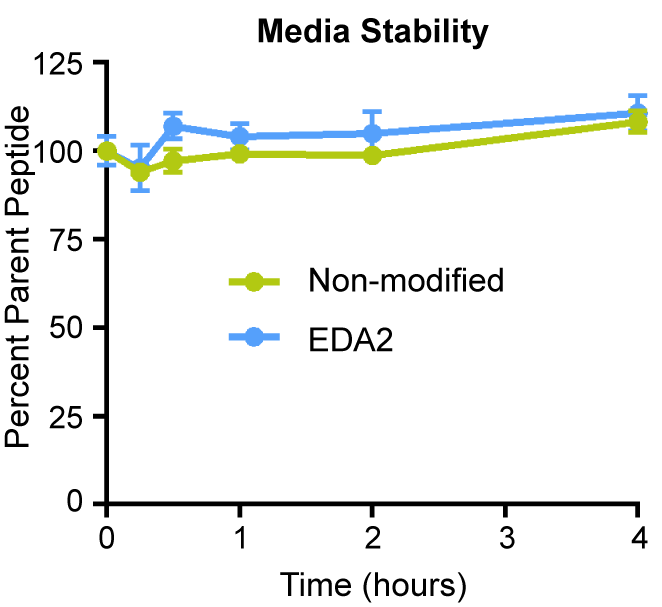

Supplement: S2 Fig — Peptide stability was measured in the presence of RPMI-1640 tissue culture medium over a time range of 0–4 hours at 37°C. The relative amount of loss as compared to that of the parent peptide at t = 0 was quantified by LC/MS using an internal standard. Data is plotted in GraphPad Prism as the average of duplicates, where error bars represent SEM. (TIF) [file pone.0118796.s002.tif]

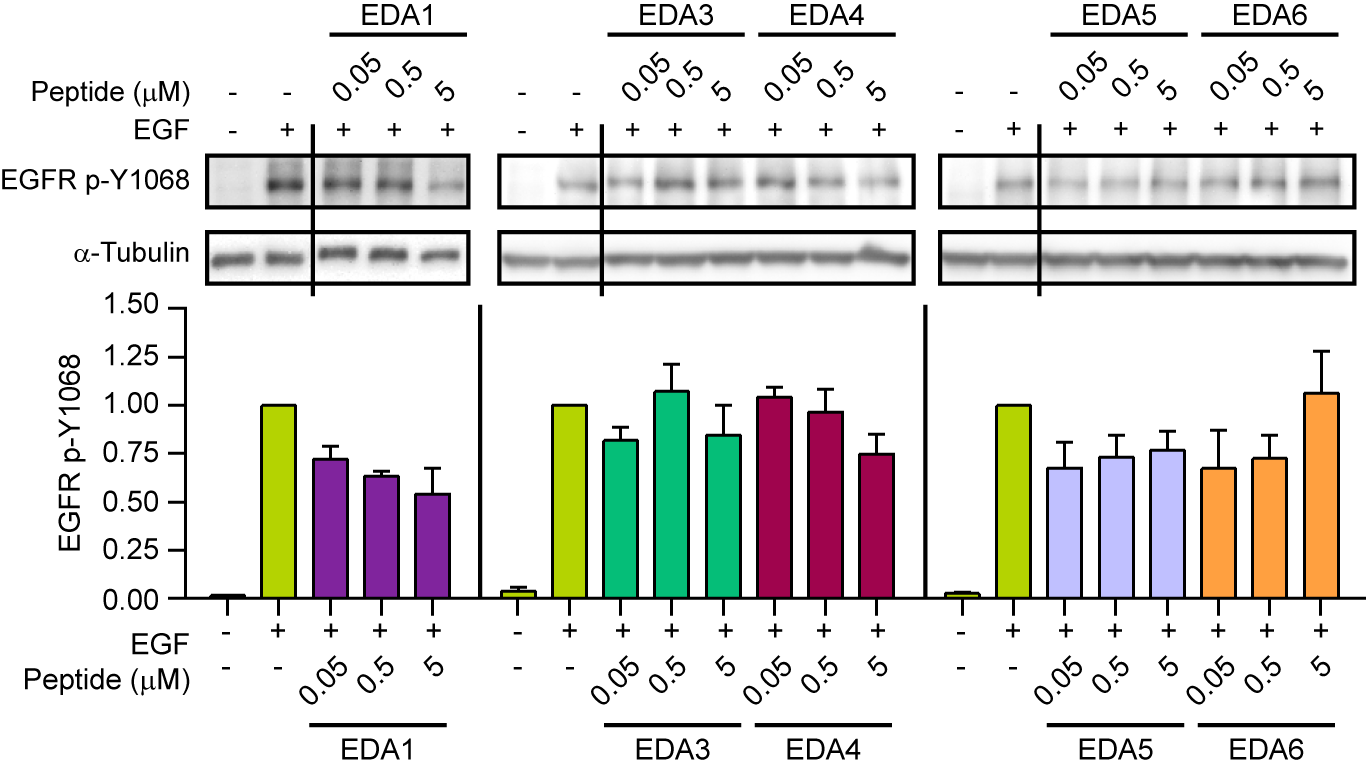

Supplement: S3 Fig — Serum starved MDA-MB-231 cells were treated with peptide or vehicle for 30 minutes, after which cells were stimulated for 5 minutes with 50 ng/mL EGF. Cells were immediately lysed following stimulation and proteins were separated by 8% SDS-PAGE. Western blot analysis showed that EDA1 and EDA3–6 do not inhibit EGFR phosphorylation at Tyr1068. Vertical lines indicate non-adjacent samples from the same western blot. Data is plotted as the average of three experiments, where error bars represent SEM. All peptide means did not significantly differ (p > 0.05) from the EGF-stimulated control. (TIF) [file pone.0118796.s003.tif]

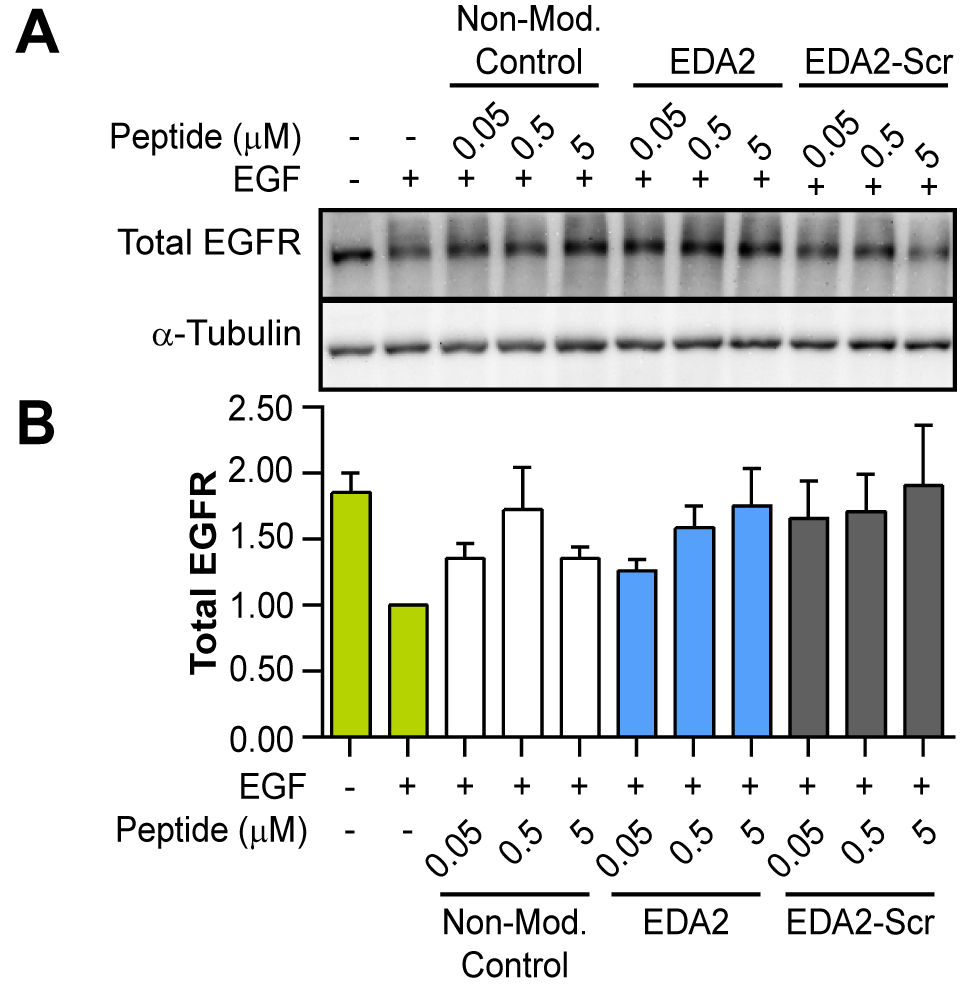

Supplement: S4 Fig — (a) Serum starved MDA-MB-231 cells were treated with peptide or vehicle for 30 minutes then stimulated with 50 ng/mL EGF for 5 minutes. Cells were immediately lysed and total EGFR levels were analyzed by western blotting. (b) Total EGFR was quantified and normalized to the tubulin loading control. Data is plotted as the average of two experiments performed in duplicate and triplicate, where error bars represent SEM. The means did not significantly differ (p > 0.05), indicating that the peptides do not affect total EGFR protein levels in the cell. (TIF) [file pone.0118796.s004.tif]
